# Duplication of uniqueness: electrotyping in nature printing and its application in contemporary art

**DOI:** 10.1186/s40494-019-0263-0

**Published:** 2019-03-29

**Authors:** Valentina Ljubić Tobisch, Albina Selimović, Anna Artaker, Martin Klobassa, Wolfgang Kautek

**Affiliations:** 1 Department of Physical Chemistry, University of Vienna, Währingerstrasse 42, 1090 Vienna, Austria; 2 Academy of Fine Arts Vienna, Elise-Richter-Research Fellow, Augasse 2-6, 1090 Vienna, Austria; 3 Sculptor and Metal Designer, Mariahilferstrasse 49/1/26, 1060 Wien, 1070 Vienna, Austria

**Keywords:** Nature printing, Printing plates, Electrotyping, Conductive layer, Heritage science, Contemporary art

## Abstract

The nature printing technique was designed for the electrotyping reproduction of leaves and other natural products. Authentic impressions could be performed by inserting leaves between two lead plates or pressing leaves into the lead plate by a press. The impression obtained in the soft lead plate could then be further reproduced by copper electroplating. Electrochemically deposited copper is hard-wearing and therefore very suitable for the production of printing plates. However, depending on the technical implementation and the choice of the materials used, decisive differences in the faithfulness of reproductions of original motifs may occur during the electrochemical deposition. A central topic in electroforming of printing plates is the choice of the conductive layer on the mould. In the present study, it has been shown that graphite powder represents a conductive phase on the siloxane mould superior to silver and copper powder. The grain size of the copper electrodeposit depended on the powder grain size. The copper plate deposited on graphite powder showed the lowest grain size (5–20 µm) and the highest homogeneity of the print background. Hand polishing of the printing plate resulted in a much better faithfulness of the motif details than that of the machine polished version. However, the background of the print derived from the machine polished plate was the most homogeneous. Electrochemical investigation showed that remnants of the silver powder could result in local elements that could enhance corrosion and thus impair the durability of the printing plates. This phenomenon was negligible with the conductive layers consisting of graphite and copper.

## Introduction

### History of nature printing

A nature print is a print in which an object taken from nature serves as a printing block. They are produced by the direct use of e.g. plants or animal objects—without interaction or artistic interpretation. Nature printing was developed in the Middle Ages to support the collections of medicinal plants in parallel to the botanical tradition of the production of herbaria. The durability of herbaria being limited, botanists started to soak the dried and pressed plants in ink to print them on paper. The first impressions of leaves and fruits obtained were very simple. The earliest dateable nature print dates to 1228. By the seventeenth and eighteenth century, nature printing had developed into a serious scientific method of plant reproduction. Nature printing was also popular in Asia, namely in Polynesia, China and Japan, where prints of fish and other sea species are still being produced [1, 2].

Historic herbal books describe that both sides of the plant were usually ink-coated [3]. The plant was then placed between two sheets of paper and pressure was applied to achieve a true-to-the-original imprint. This process could be repeated up to two more times without requiring a renewed soaking of the object in additional ink. Usually six prints could be obtained using the same plant. While the first two prints generally turned out rather dark, the subsequent two prints were the most faithful duplicates. The last two prints often had to be coloured once they had dried out, while the truest second ones were left uncoloured. In the sixteenth century, several European botanists knew of and applied the technique which was known as *ectypa* or *typographia plantarum* at the time [2, 3].

The first step in the process of nature printing, the technique developed and patented by Alois Auer and the Austrian National Printing Office (k. k. Hof- und Staatsdruckerei) in 1852 [4] was the creation of an original imprint of the object in lead by means of a printing press. From this original imprint, a copper intaglio printing plate was produced using the process of galvanoplasty or electrotyping, which was a cutting edge technology at the time [5]. The time-consuming electrotyping process was employed twice. First a positive of the imprinted plant was made from the negative impression in lead [6]. This positive served as a matrix for a second plate produced in a galvanic copper bath, which was identical to the initial imprint in lead, but suitable as a printing plate. When the quality of the copper printing plate started to deteriorate a new one could be produced from the matrix. In principle, the process enabled a theoretically endless print run, although in practice the laboriousness involved prevented large editions [7, 8].

**Fig. 1:**
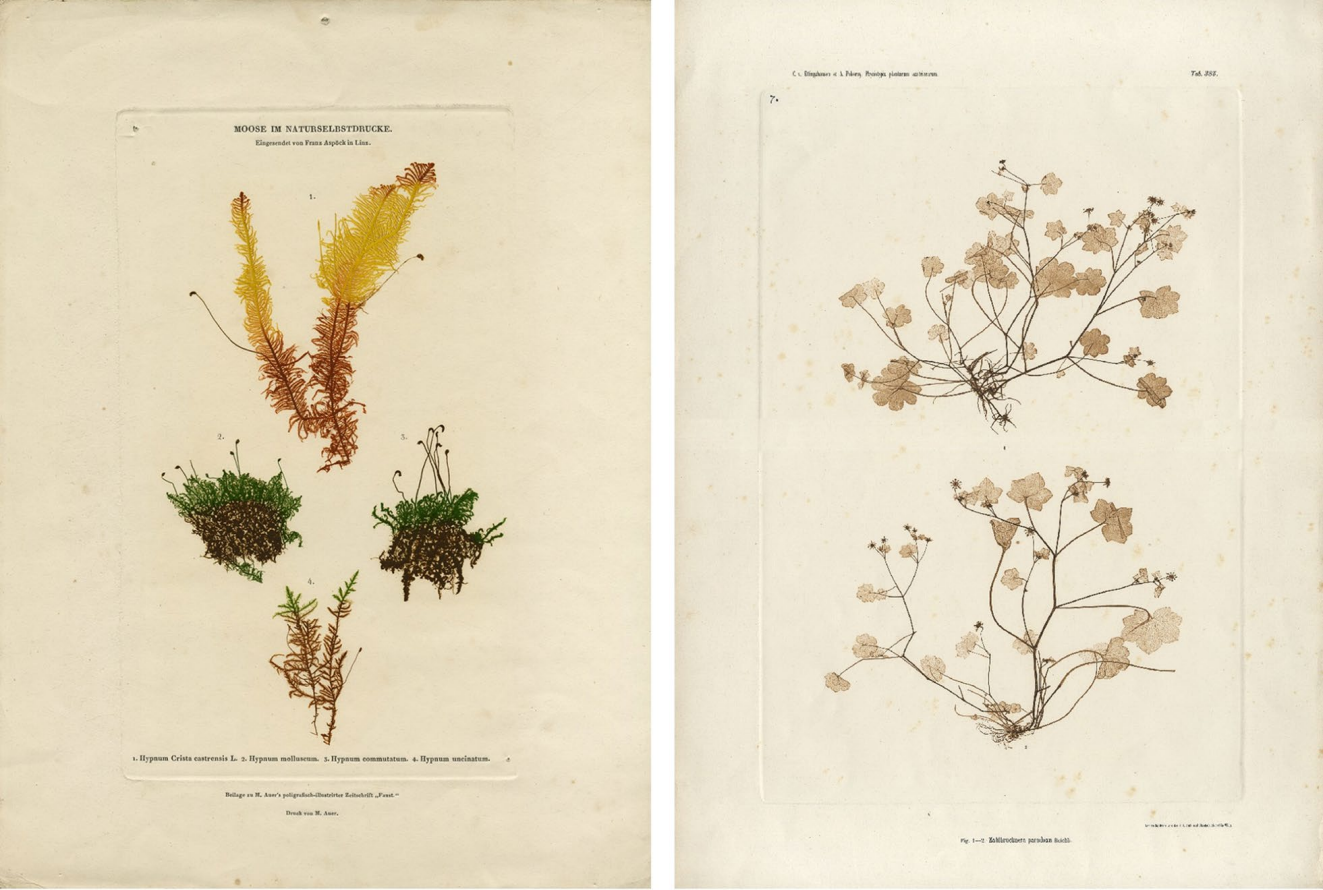
Left: nature print of spondulicks, 1854, Franz Aspöck, (M. Auer, Faust, Poligrafisch-illustrirte Zeitschrift, 1854, Vienna) © Technisches Museum Wien. Right: nature print of Zahlbrucknera, Ettingshausen & Pokorny, k.k. Hof- und Staatsdruckerei, 1856, Vienna, © Technisches Museum Wien

This technique allowed the production of printing plates from dried plants, fabrics, lace samples, embroideries and other originals. It enabled the production of impressions “identical to the original”, in Auer’s words, both in white colour on dyed ground and in natural colours on white paper, even with the use of delicate embossments and recesses. Two examples are represented in Fig. 1. If we consider the touch of the plant that leaves an imprint a unique and irretrievable moment, then the Viennese technique of nature printing enables the paradoxical duplication of uniqueness. The main motive for the development and perfection of this printing technique by the Austrian National Printing Office was the possibility of its application in the production of large editions and its interest in duplicating processes.

### Contemporary attempt to recreate the historic technique

One of the authors, the artist Anna Artaker combines her interest in the history of photography and William Henry Fox Talbot’s earliest botanical photograms with a revival of the historical technique of nature printing. She presented a series of contemporary nature prints entitled “The Pencil of Nature” at the Bank Austria Kunstforum in Vienna, Austria, in 2017 [9]. If nature is defined as encompassing all phenomena that occur without human intervention, is it possible to reproduce them without changes? Considering that the touch of the plant leaves an impression of a unique and irretrievable moment, the Viennese technique of natural self-printing allows the paradoxical doubling of uniqueness.

**Fig. 2:**
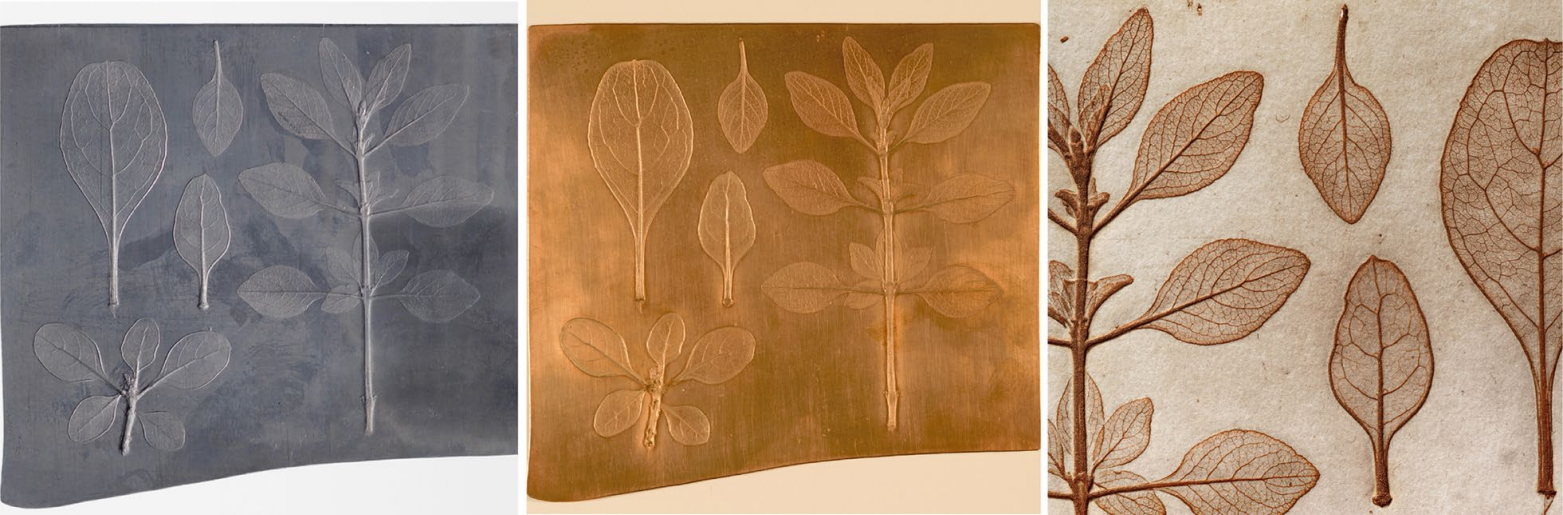
Photographs of the lead plate onto which the plant was impressed (left), of the copper plate, the positive (middle), of the print on paper (right). © Anna Artaker

While trying to reconstruct the nature printing technique of Alois Auer, questions and problems concerning the surface quality of the produced prints arose. A graininess in the plate background was observed that should appear as smooth and flawless as possible to bring the motif to the foreground. The resulting print exhibits its own base tone originating from the graininess of the matrix (Fig. 2).

Electrochemical deposition was used fairly quickly after its discovery at the end of the 1830s both for artistic and multiplication purposes [5]. If the matrix material was not electrically conductive, a coating process with an electrically conductive material was necessary. This was achieved either by the application of electrically conductive powders or by electroless metal deposition [10]. The main requirement for the conductive layers is that it generates a thin film imperceptible enough, so that it does not impair the shape or the details of the motifs to be replicated. The cheapest and most commonly used method in historic techniques was graphitizing. Apart from finest graphite powder, metal powders such as silver, copper or even gold powder were used [11–13].

The present investigations intended to provide information on the electrical conductivity of graphite, silver and copper powders on the one hand and on the quality and roughness of the electroplated surface on the other hand [14]. The experiments allowed to identify the optimum electrode material for the most ideal moulding and showed whether parts of the conductive layer were transferred to the electroformed positive.

Electrochemical methods of analysis play an increasingly important role in conservation science [15, 16]. Particularly advantageous is the fact that they are practically non-destructive methods. In fact, most electrochemical methods of analysis can be performed directly on the object without the necessity of sampling. The size and shape of the object do not play a major role since the electrochemical methods are transportable and can be applied in situ. They can be used to evaluate the protective properties of coatings on metal surfaces in the field of heritage science [17]. Besides the use of electrochemical methods in the analysis of metal objects, the electrolytic reduction of tarnish layers on metals is an established method of intervention in the restoration of metal artefacts [15].

The main task of this study was a comparison of three conductive layers which have historically been used in the electrochemical deposition of copper. Conductive layers are believed to play an important role in the faithful reproduction of such impressions. A considerable amount of research has been invested in the historical studies of natural self-printing technology, but no known study has yet focused on the microscopic surface of the printing plates. Moreover, cyclic voltammetry was employed to identify the components of the surface layers and their corrosion stability on the produced copper plates as well as the effects of various surface polishing treatments after the electrotyping process.

## Experimental

Production of electrotype samples started with the impressing of plant motives onto a 3 mm thick lead plate. A negative of this plate was produced using a two-component siloxane (MM 240 RTV, Bodo Möller Chemie Austria GmbH) and was mounted on a stable support plate of acrylic glass. One-third of the siloxane surface was coated with copper powder (Gold Bronze Powder, Eckart-Werke, Germany), the second third with graphite powder (Graphite Fine Powder extra pure, Merck, Austria) and the last third was coated with silver powder (Silver Powder 1000/1000, Ögussa, Austria). The metal powders exhibited platelet morphologies resulting from their electrolytic production. All three powders were used as received by the manufacturer. They were first applied with a soft brush and then smoothed into the siloxane surface with fingers. The excess was removed with compressed air. The application of the conductive powders were originally carried out with a long-haired brush [10]. The brush was dipped into the conductive powder and led in circular movements over the negative mould until the whole surface showed a metallic shine.

**Fig. 3:**
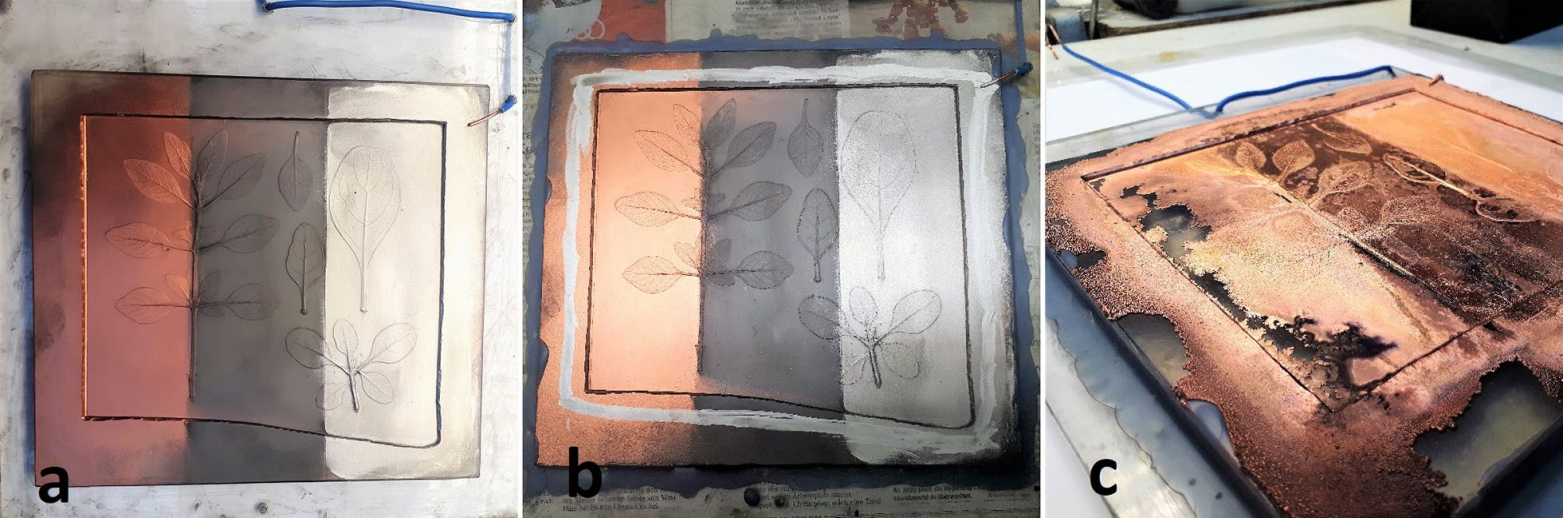
Siloxane mould. **a** Left section coated with Gold Bronze Powder 9900 Copper; middle section coated with Graphite Fine Powder extra pure; right section coated with Silver Powder 1000/1000. **b** Powder coated sample with additional contact silver lacquer electrode and with wax seal to prevent electrolyte penetration. **c** Electrodeposited copper layers

The conductive layers on the siloxane mould were electrically contacted by an insulated copper wire (Fig. 3a). This wire also served as a mechanical suspension device in order to hold the acrylic base and the mould in the electrolytic bath. An additional contact silver lacquer electrode was applied along the circumference of the sample (Fig. 3b). The space between the acrylic glass plate and the siloxane mould was sealed with liquid wax to prevent the electrolyte from penetrating in between.

This sample was submersed into a sulphuric acid thick-film copper bath (Bright Copper ACG 8, Schlötter Galvanotechnik) at 20 °C (Table 1). This acid copper electrolyte contains a single additive not disclosed by the manufacturer. The plated deposit has a high deposition rate, very low internal stress and is soft which makes polishing very easy. The bath was kept in constant motion by a pump. A DC-power supply (Heraeus, 10 V, 100A) served as current source for the electrolysis. The copper anodes (200 × 400 mm, 1 cm thick) were enclosed by polypropylene anode bags. A voltage of 0.5 V was applied until the entire sample surface was covered. Then, it was increased to 2 V. After 4 h, the negative plate (working electrode) was rotated by 180° and the electrolysis was continued another 4 h. After this electrodeposition process, the deposited copper plate (Fig. 3c) was separated from the underlying mould with a blunt knife which was inserted between the siloxane negative and the copper positive. The copper plate was rinsed with water and buffed with a soft brass wire brush, as described in the historical reports [11–13].

**Table 1:** Galvanic bath “Glanzkupferbad ACG 8”

Parameter	Information provided by manufacturer
Cu concentration	39.0 gl^−1^
H_2_SO_4_ concentration	97.8 gl^−1^
Cl^−^ concentration	58.0 g^−1^
Fe concentration	0.1 gl^−1^

Polishing of the galvanic copper printing plates has been an established finishing step. Mechanical polishing of the buffed and brushed sample removed various roughnesses on the surface by pressing and rubbing so that the individual asperities were eliminated until the surface appeared mirror-smooth. Historically, various tools have been used, namely polishing steel, agate, some types of horn and bloodstone/hematite [12]. Thin galvanic layers were often polished with leather which was coated with a mixture of oil and a hard powder such as pumice stone, tin ash or bloodstone. Later, galvanic objects were polished on the polishing machine with a rotating brush or fabric disc.

It is not clear how Alois Auer polished the obtained printing plates for the production of nature prints. Therefore, in the present work, three surface states were examined. The plate segments, made conductive with silver, graphite and copper powder respectively, were sawn into three segments so that each contained one-third of each powder type. One-third was polished manually using bloodstone while the second third was polished with a fabric disc on a polishing machine and the last third remained untreated (compare Fig. 6 below). A polishing paste was used with a rotating fabric disc (Baier’s Enkel Nfg., Silberglanz-Paste, fat-bound).

Measuring windows of ca. 1 cm^2^ serving as working electrode surfaces were generated on each plate segments by an electrically insulating siloxane adhesive (Saint-Gobain Performance Plastics MG Silicon GmbH, SK 6000 Neutral). Electrochemical measurements were carried out with an Ag/AgCl reference electrode (RE, + 0.222 V vs. SHE [18] and a gold counter electrode in the form of a wire spiral (CE) in a glass tube with a porous frit as electrolyte junction. The cyclic voltammetry and the open circuit transient measurements were performed with an electrochemical workstation (CH Instruments, 760C). A 0.1 M K_2_CO_3_ (pH = 10.6) solution with deionized water (Milli-Q) served as electrolyte. The pH value was determined by a glass membrane electrode (Metrohm, 780 pH Meter). Before starting the electrochemical measurements, the plate was degreased inside the measuring window by wiping with a cotton swab soaked with acetone.

Optical micrographs were recorded with a digital microscope (Keyence, VHX-5000). Electron micrographs were registered with an environmental scanning electron microscope (FEI, Quanta 250 FEG with a Schottky field emission source).

## Results and discussion

The metal powders applied on the insulating siloxane moulds constitute the conductive electrodes for the electrodeposition of the copper layers. The morphology, the volume resistivity, and the thickness of the applied metal powders should have an influence on the quality of the electrotypes.

**Fig. 4:**
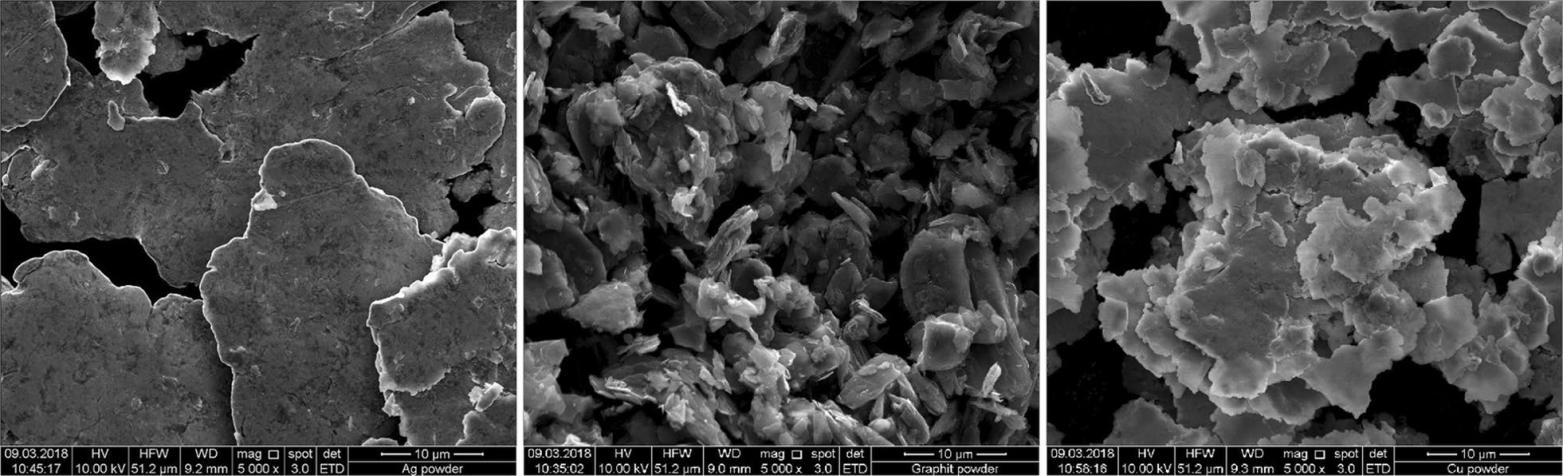
Scanning electron micrographs of Silver Powder 1000/1000 (left), Graphite Fine Powder extra pure (middle) and Gold Bronze Powder 9900 Copper (right). 10 kV, ×5000

**Fig. 5:**
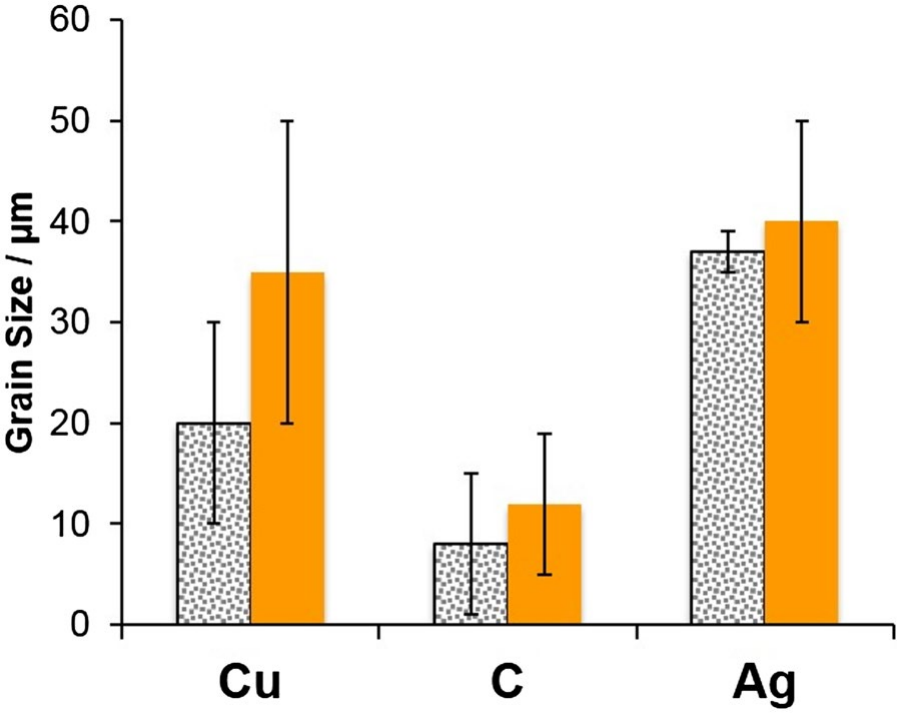
Conductive powder (dotted) and electrodeposited copper plate (orange) grain sizes

The local current density affects the deposit morphology [19]. It is proportional to the reciprocal of the volume resistivity and the thickness of the applied powder layer at an applied constant potential:

1 $$j \propto 1/R \propto 1/\rho \times d$$

with the powder layer thickness *d*, resistance R, and the volume resistivity ρ (Eq. 1). The respective ρ are represented in Table 2. ρ of graphite exhibits the highest value in respect to Cu and Ag. The powder grain sizes and the Cu deposit grain sizes were evaluated by scanning electron microscope (SEM; Fig. 4) and optical microscopy, respectively. According to Eq. 1, graphite should exhibit the lowest local current density at a *d* ≈ 50 µm. The lower the current density the higher the contribution of the base reproduction growth type versus the field-oriented type will be. This correlation led to a smooth and small-grained deposit for graphite (Fig. 5, Table 2).

**Table 2:** Conductive powder properties and electrodeposited copper plate grain sizes

Name	Volume resistivity, ρ [Ωm], 20 °C	Powder layer thickness *d* [μm]*	Powder grain size [μm]**	Cu deposit grain size [µm]*
Cu (gold bronze powder 9900)	1543·10^−8^ (18)	55.6 ± 8.4	10–30	20–50
Graphite (graphite fine powder extra pure)	3500·10^−8^ (18)	47.5 ± 7.0	1–15	5–20
Ag (silver powder 1000/1000)	1467·10^−8^ (18)	55.2 ± 17.9	35–40	20–50

* Evaluated from optical micrographs. **Evaluated from scanning electron micrographs

When a base reproduction occurs at moderate inhibition intensity by an additive, the respective powder morphology can have a direct influence on the electrodeposit texture. The base electrode graphite shows the smallest grain size (< 15 µm) whereas Ag shows greater sizes (> 35 µm), and Cu a broad distribution of up to 30 µm (Figs. 4 and 5, Table 2). Therefore, the observed Cu deposit grain size on graphite resulted in the lowest value (Figs. 4 and 5, Table 2).

**Fig. 6:**
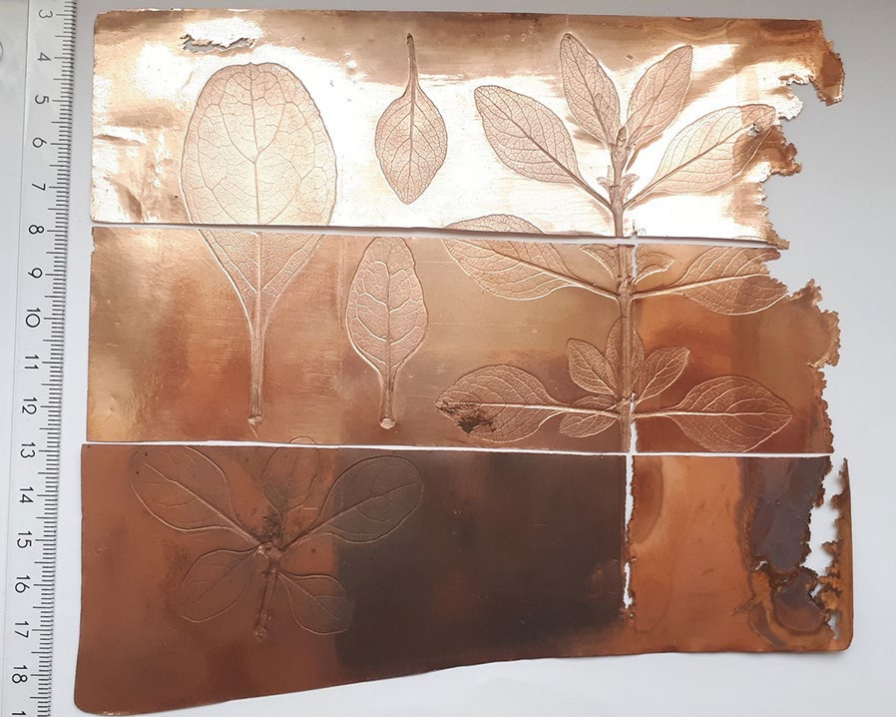
Photograph of the electrodeposited copper printing plate. Vertical column with various conductivity powders on siloxane. Left: Ag. Middle: graphite. Right: Cu. Horizontal rows with various post treatments. Bottom: untreated. Middle: machine polished. Top: hand polished

The Cu plate electrodeposited on Cu powder was an incomplete, uneven, and inhomogeneous layer (Figs. 3c and 6). The region near the inhomogeneities were partially delaminated and exhibited darkened and “burned” surfaces. Therefore, electrolyte penetrated also between the Cu deposit and the Cu powder layer on siloxane mould. The Cu edges showed grainy and powdery morphology due to enhanced local current densities. The “burned” surface features can be correlated with high rates of hydrogen discharge [20]. This leads to a pH increase in the interface resulting in the precipitation of hydroxide or basic salts of Cu. These may be occluded by the plated deposit.

**Fig. 7:**
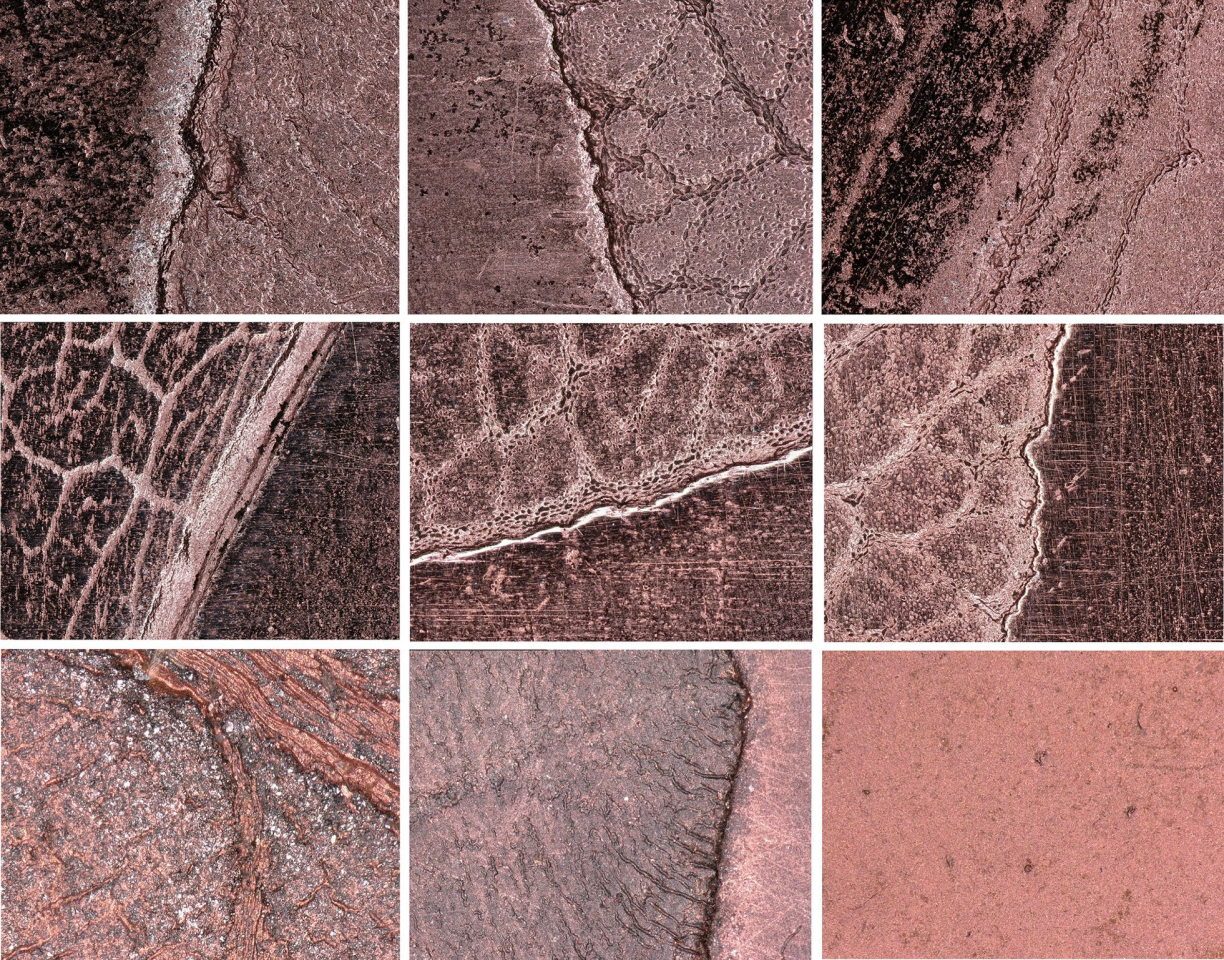
Optical Micrographs of the moulded side of the electrodeposited copper printing plate. Vertical column with various conductivity powders on silicon. Left: Ag. Middle: graphite. Right: Cu. Horizontal rows with various post treatments. Bottom: untreated. Middle: machine polished. Top: hand polished

The Cu plate electrodeposited on either graphite or Ag powder provided a homogeneous and compact layer (Fig. 6). Powder remnants still adhered visibly in depressions (Fig. 7, Table 3).

**Table 3:** Copper deposit characterization

Conductive layer	Untreated	Machine polished	Hand polished
Cu (gold bronze powder)	Incomplete, uneven, inhomogeneous, burned. Matt appearance	Incomplete, uneven, inhomogeneous plate. Scratches on elevations. Poor faithfulness to the original	Incomplete, uneven, inhomogeneous plate. Smoother than machine polished, no scratches. Moderate faithfulness to the original
Graphite powder	Complete, compact layer. Matt appearance Remnants of graphite powder	Scratches on elevations. Impaired faithfulness to the original	Smoother than machine polished, no scratches. Best faithfulness to the original
Ag (conductive silver powder)	Complete, compact layer. Matt appearance Remnants of Ag powder	Scratches on elevations. Poor faithfulness to the original	Smoother than machine polished, no scratches. Moderate faithfulness to the original

The after treatment was performed by hand (stone) polishing using hematite (bloodstone) and machine polishing (rotating fabric disc). The advantages of stone polishing are the higher gloss, the texture of the surface becomes denser, and the metal loss is minimized in contrast to machine polishing. The elevated parts of the surface profile appeared dark in the machine polished regions. This is due to the specular reflection of the illumination light which cannot be collected in the microscopic objective. Fine one-directional scratches were introduced in these flat regions by the rotating fabric disc treatment. Hand polishing yielded much shinier regions without the leaf motifs than machine polishing. Practically no scratches were introduced by the hematite/bloodstone treatment. Hand polishing did not affect the depressed features of the original leaf in contrast to machine polishing. A substantial difference in quality was observed between the electroformed leaf details of the plate deposited on Ag and graphite: the faithfulness to the original was by far superior on the Cu plate deposited on the graphite base electrode than that deposited on the Ag base electrode. A Cu base electrode resulted in a rough surface. Machine and hand polishing yielded comparable qualities as the Cu plate deposited on the Ag base electrode.

**Fig. 8:**
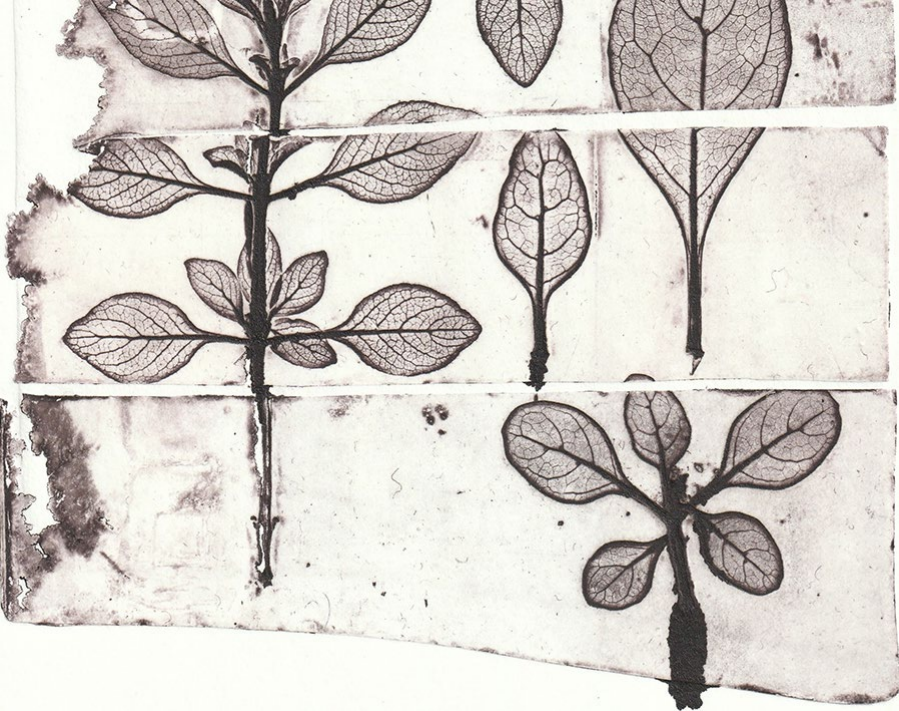
Photograph of the print on paper derived the plate shown in Fig. 6. Vertical column with various conductivity powders on siloxane. Left: Cu. Middle: graphite. Right: Ag. Horizontal rows with various post treatments. Bottom: untreated. Middle: machine polished. Top: hand polished

The prints of the three plates separated by their polishing treatment (Fig. 6) showed drastic differences in quality (Fig. 8). The faithfulness to the original leaf motif from the hand polished plate was by far superior to that treated by machine polishing. On the other hand, the background of the print from the machine polished plate was more homogeneous than that from the hand polished. The print from the untreated plate exhibited a good faithfulness to the original leaf motif but showed strong inhomogeneities of the background.

The remnants of the conductive powder layers on the electroformed Cu printing plate introduce local elements which can accelerate localized corrosion reactions. This can yield enhanced roughness thus impairing the long-term stability and applicability of such artefacts. The faithfulness of prints would be diminished by an increased plate roughness. Also, the graininess in the plate background would be enhanced.

**Fig. 9:**
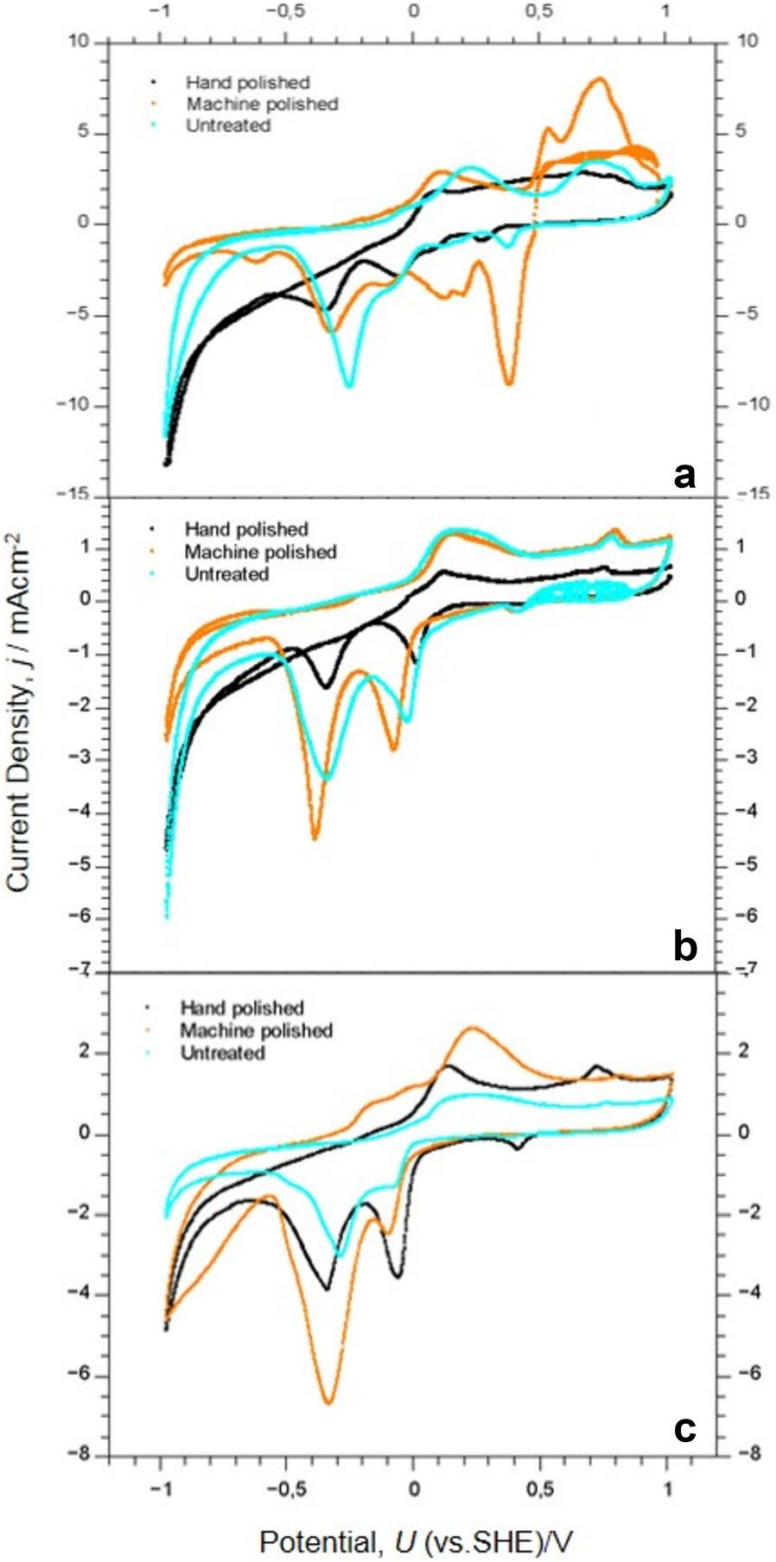
Cyclic voltammograms of copper plate surface in 0.1 M K_2_CO_3_, pH = 10.60. Scan rate = 10 mVs^−1^. Comparison of **a** silver, **b** graphite, **c** copper powders as conductive layers on the mould. Parameters: polishing methods

The corrosive behaviour was studied by cyclic voltammetry in a 0.1 M K_2_CO_3_ solution (pH = 10.6). The Cu oxidation to Cu(I) oxide

2 $$2 {\text{Cu }} + {\text{ H}}_{ 2} {\text{O}} \rightleftharpoons {\text{Cu}}_{ 2} {\text{O }} + {\text{ 2H}}^{ + } + {\text{ 2e}}^{ - }$$

and the further oxidation to Cu(II) oxide

3 $${\text{Cu}}_{ 2} {\text{O }} + {\text{ H}}_{ 2} {\text{O}} \rightleftharpoons 2 {\text{CuO }} + {\text{ 2H}}^{ + } + {\text{ 2e}}^{ - }$$

as well as the respective reduction reactions could be detected with remnant Ag, graphite powder, and Cu powder even after machine and hand polishing (Fig. 9). The anodic peak of the Cu_2_O formation appeared at a potential positive of the Nernst equilibrium potential of this reaction *U*_*0*_ = − 0.16 V (Eq. 2, Table 4). The reduction of Cu_2_O occurred as a peak between ca. − 0.2 and − 0.4 V. The oxidation of Cu_2_O to CuO supported a broad anodic current wave at *U* > + 0.5 V (Eq. 3, Table 4). The reduction of CuO yielded a peak negative of the respective Nernst equilibrium potential *U* = + 0.04 V. Remnant Ag powder shows a strong redox activity and thus introduced a local element. The Ag oxidation on an untreated and a machine polished sample led to anodic peaks at *U* > 0.5 V. The first may be related to the oxidation to Ag^+^ and the second peak around +0.7 V which is positive of the Nernst equilibrium potential *U*_*0*_ = + 0.54 V is correlated with the oxidation to silver (I) oxide (Table 4).

**Table 4:** Reactions of copper in alkaline solution (21)

Reaction	Nernst equilibrium potential vs. SHE, *U*_*0*_	Nernst equilibrium potential vs. SHE, *U*_*0*_ pH = 10.60
2Cu + H_2_O ⇌ Cu_2_O + 2H^+^ + 2e^−^	0.47–0.059 pH	− 0.16
Cu + H_2_O ⇌ CuO + 2H^+^ + 2e^−^	0.57–0.059 pH	− 0.06
Cu_2_O + H_2_O ⇌ 2CuO + 2H^+^ + 2e^−^	0.67–0.059 pH	+0.04
Ag ↔ Ag^+^ ⇌ e^−^	0.80 + 0.059 log[Ag^+^]	~ + 0.50 ([Ag*] ~ 10^−5^ M
2Ag + H_2_O ⇌ Ag_2_O + 2H^+^ + 2e^−^	1.17–0.059 pH	+0.54

4 $$2 {\text{Ag }} + {\text{ H}}_{ 2} {\text{O}} \rightleftharpoons {\text{Ag}}_{ 2} {\text{O }} + {\text{ 2H}}^{ + } + {\text{ 2e}}^{ - } .$$

The reduction of Ag_2_O yielded negative peaks at U < *U*_*0*_ = + 0.54 V. Machine polishing led to a strongly scratched surface resulting in an increased surface area and higher current densities. The hand polished and untreated surfaces exhibited drastically smaller redox current densities.

These results demonstrate the strong electrochemical activity of Ag powder remnants, even after polishing treatments. Therefore, a local element of this noble metal can cause the dissolution and corrosion of the Cu printing plate. It must be pointed out that hand polishing with hematite/bloodstone resulted in the lowest electrochemical activity in contrast to machine polishing.

## Conclusions

The nature printing technique, originally developed in the nineteenth century, makes use of plants and other natural subjects to produce images. In the present study, synergistic effects of the cooperation between contemporary art and heritage science in the reconstruction of the historical technique of nature printing are presented. New plates for nature printing were produced using the historic process in order to gain a better understanding of the production technique. The aim was to make individual production steps comprehensible and to find practicable solutions for difficulties encountered in production. One of the main challenges was the production of electrodeposited copper printing plates usually preceded by the coating of the motif with an electrically conductive material. Three different conductive layers, copper, graphite and silver powders, were investigated in respect to their efficacy by morphological investigations using electron microscopy, optical microscopy, and cyclic voltammetry. The suitability of the electrochemically deposited printing plates could be related mainly to the various grain sizes and the electrical conductivities of the investigated conductive powders, as well as the polishing treatments. It could be confirmed that graphite is the best electrochemical conductor in the manufacture of printing plates with high faithfulness to the original motif.

## Abbreviations

*d*: layer thickness
DC: direct current
CE: counter electrode
*j*: current density
*l*: litre
*g*: gram
NHE: normal hydrogen electrode
pH: power of hydrogen
*R*: resistance
ρ: volume resistivity
RE: reference electrode
SEM: scanning electron microscope
SHE: standard hydrogen electrode
